# Differences in Arbuscular Mycorrhizal Fungal Community Composition in Soils of Three Land Use Types in Subtropical Hilly Area of Southern China

**DOI:** 10.1371/journal.pone.0130983

**Published:** 2015-06-24

**Authors:** Caihuan Wang, Zhenhong Gu, Hang Cui, Honghui Zhu, Shenlei Fu, Qing Yao

**Affiliations:** 1 South China Agricultural University, College of Horticulture, Guangzhou, 510642, PR China; 2 Guangdong Institute of Microbiology, Guangzhou, 510070, PR China; 3 Key Laboratory of Vegetation Restoration and Management of Degraded Ecosystems, South China Botanical Garden, Chinese Academy of Science, Guangzhou, 510160, PR China; IPK, GERMANY

## Abstract

Land use type is key factor in restoring the degraded soils due to its impact on soil chemical properties and microbial community. In this study, the influences of land use type on arbuscular mycorrhizal fungal (AMF) community and soil chemical properties were assessed in a long-run experimental station in subtropical hilly area of southern China. Soil samples were collected from forest land, orchard and vegetable field. Soil chemical properties were analyzed, and PCR-DGGE was performed to explore the AMF community structure. Cloning and sequencing of DGGE bands were conducted to monitor AMF community composition. Results indicate that the contents of total P, available P and available K were the highest while the contents of soil organic matter, total N, total K and available N were the lowest in vegetable field soils, with forest land soils *vice versa*. According to DGGE profiling, AMF community in forest soils was more closely related to that in orchard soils than that in vegetable field soils. Sequencing indicated that 45 out of 53 excised bands were AMF and 64.4% of AMF belonged to Glomeraceae, including some “generalists” present in all soils and some “specialists” present only in soils of particular land use. Category principle component analysis demonstrated that total N, soil organic matter and available P were the most important factors affecting AMF community, and some AMF phylotypes were closely associated with particular soil chemical properties. Our data suggest that AMF communities are different with different land use types.

## Introduction

Southern China is one of the most populated regions, resulting in strong pressure on soil environments. In this region, the typical agricultural soils are red soil [1], classified as Ultisols in the Soil Taxonomy System of the USA and Acrisols and Ferralsols in the FAO legend [2]. Dominated by hilly or mountainous geomorphology and monsoon climate, this region is characterized by severe soil erosion, especially in the rainy season at the intensive farming areas [3–5]. To restore the eroded soils, many investigations have focused on the potential of land use types to decrease the soil erosion and positive results have been reported repeatedly [4], [6]. Presently, the most popular land use types in this region include forest lands, orchards and sometimes vegetable fields [4], [5], [7], representing low, moderate and high intensity of land use, respectively. On the other hand, the feedback of soil environments to land use types is of special interest to soil ecologists. The effects of land use types on soil erosion and soil physiochemical properties have been intensively evaluated [8–11], however, information of the effects on soil microbial community, especially arbuscular mycorrhizal fungal (AMF) community, is less reported.

Soil microbes are essential biotical components in soil ecosystems, involved in almost all biogeochemical processes in pedosphere [12], [13]. Taking AMF, a kind of symbiotic soil fungi, as example, they normally establish symbiosis with >80% terrestrial plants [14], and actively help host plants resist to diverse environmental stresses [15], with surprising effect on nutritional stress [16]. In degraded soils, nitrogen (N) and phosphorus (P) are main limiting factors for well ecosystem functioning, and thus, inputs of N and P to are of primary significance for restoration of the degraded ecosystems [17]. In this circumstance, AMF may play a unique role in it. There is some evidence that N-uptake is enhanced by some AMF [18–21]. The promotive effect of AMF in plant P uptake has been more intensively and deeply investigated and confirmed for decades [14]. In southern China, AMF species are abundant partially due to the diverse plant species in this region, with higher diversity than that in the northern China [21]. It is also established that AMF in forest lands are diverse on the basis of both morphological types and genetic types [22], [23], which lays a pavement to the utilization of AMF in restoring the eroded soils in the hilly or mountainous areas in southern China. On the other hand, however, land use types may shift AMF community [24–26], leading to uncertain ecological consequence. For example, Oehl et al. [24] demonstrated that AMF species richness decreased with increased land use intensity, which supported by Tchabi et al. [27]. In contrast, unchanged and even increased AMF species richness have also been reported [25], [26]. Clearly, when different land use types are practiced in the hilly or mountainous areas in southern China, not only the full use of AMF but also the protection of AMF resource should be born in mind. Unfortunately, up to date, no information on the effects of land use types on AMF community in this region is available.

In this study, we chose a long-run ecological experimental station, Heshan Hilly Land Interdisciplinary Experimental Station of the Chinese Academy of Sciences, as experimental sites to explore: i) the AMF resource and diversity; ii) the effects of land use types on the AMF community; and iii) the possible mechanisms underlying the effects in the restored subtropical soils in southern China. In this experimental station, much data has been accumulated involving diverse ecological processes, however, without any data on AMF community.

## Materials and Methods

### Site Description and Soil Sampling

This study was carried out at the Heshan Hilly Land Interdisciplinary Experimental Station (HHLIES), Chinese Academy of Sciences (CAS), China, and was permitted by HHLIES. The climate in this region is typical of subtropical monsoon. The average annual temperature and precipitation are 21.7°C and 1700 mm, respectively [28]. Studies involving multiple disciplines have been conducted in this station to explore the ecosystem processes in the subtropical areas [28–33].

Three land use types, e.g. vegetable field (112.9045°E, 22.6720°N), orchard (112.9053°E, 22.6722°N) and forest land (112.9052°E, 22.6717°N) (representive of land use with different intensity in descending order), were selected as sampling sites. In detail, four plots (5 m × 10 m) were established for each land use type, with a distance of about 20 m between plots. All plots were located at the same altitude. In each land use type, the four plots distributed in a small area, and consequently the differences among four plots represented experimental error. However, the differences among three land use types can still reflect the influence of land use type on AMF community composition. In forest land, the understory vegetation was dominated by *Dicranopteris dichotoma*, with the coverage as high as almost 100% [28]. Orchard and vegetable field were transformed from forest land and managed for over 30 and 10 years, respectively. The orchard floor was naturally dominated by *D*. *dichotoma*, while rotation cropping was practiced for vegetable production with tomato, pepper, lettuce and winter fallow sometimes. In each plot, six soil cores of 20 cm depth were randomly sampled and mixed well by sieving through 2 mm mesh. A total of 12 soil samples were collected, and each soil sample was further divided into two subsamples for the analysis of soil chemical properties and molecular analysis of AMF community.

### Measurement of Soil Chemical Properties and AMF Spore Extraction

Soils were air dried and sieved for chemical analysis [31]. Soil pH was measured in deionised H_2_O (1:2.5 w/v). Soil organic matter content was determined using the K_2_Cr_2_O_7_ titration method after digestion. Total nitrogen (TN), total phosphorus (TP) and total potassium (TK) were analyzed using Kjeldahl method, molybdenum blue colorimetric method and flame photometer, respectively. Available N (alkali-hydrolyzable N) was released and transformed to NH_3_ by 1.07 M NaOH and FeSO_4_ powder at 40°C for 24 h, and then absorbed with 2% (w/v) H_3_BO_3_ and titrated with 0.005 M H_2_SO_4_. Available P was extracted with the solution of Bray-1 (0.03 M NH_4_F-0.025 M HCl) and measured by colorimetry. Available K (exchangeable K) was extracted with 1.0 M NH_4_OAc (pH = 7.0) and then determined by flame photometer.

Twenty five gram of air dried soils were used for AMF spore extraction with the modified method of soil sieving and sucrose centrifugation [24]. The extraction followed the described procedure except that soils were rinsed in tap water for 60 min with stirring at an interval of 20 min and then the soil suspension was sonicated for 30 s before soil sieving. These steps were included to release the spores associated with soil matrix. The spore number in each sample was recorded and AMF species were identified on the basis of spore morphology under a stereo-microscope (Motic BA410) according to online information on the species descriptions (http://invam.wvu.edu/the-fungi/species-descriptions).

### Extraction of Soil Total DNA and PCR-DGGE

To extract soil total DNA, PowerSoil DNA Isolation Kit (MO BIO Laboratories Inc.) was used according to the manufacturer protocol. After soils were transported to the laboratory, soil DNA was extracted immediately, and then the extracted DNA was stored at -20°C for PCR-DGGE analysis.

To investigate the genetic diversity of AMF, 18S rRNA fragments of AMF were amplified using nested PCR strategy. The first PCR reaction with the universal eukaryotic primers NS1/NS4 amplifies a 1100 bp fragment [34], with the following PCR condition: 3 min initial denaturation at 94°C; followed by 30 cycles of 30 s at 94°C for denaturation, 1 min at 40°C for annealing, and 1 min at 72°C for extension; and a final extension at 72°C for 10 min. Products of the first PCR reaction were diluted 100-fold and used as template DNA in a second PCR reaction performed using the AMF-specific primers AML1 and AML2 [35]. The second PCR reaction was carried out with the following PCR condition: 3 min initial denaturation at 94°C; followed by 30 cycles of 1 min at 94°C for denaturation, 1 min at 50°C for annealing, and 1 min at 72C for extension; and a final extension at 72°C for 10 min. Products of the second PCR reaction were diluted 200-fold and further subjected to the third PCR reaction using primer set NS31-GC/Glo1 [36]. PCR reaction was performed with the following PCR condition: one cycle of 2 min at 94°C for initial denaturation; followed by 30 cycles of 45 s at 94°C for denaturation, 45 s at 54°C for annealing, and 35 s at 72°C for extension; and a final extension at 72°C for 10 min. The sequences of three primer sets are shown in Table 1. For each step, the PCR product was checked for correct size by electrophoresis on a 2% agarose gel stained with Gold view (SBS Genetech Inc., China). The final products were subjected to DGGE analysis.

**Table 1:** Sequences of primer sets used in this study.

Primers	Sequence (5'→3')	Amplified fragment	References
NS1	GTA GTC ATA TGC TTG TCT C	1100 bp	[34]
NS4	CTT CCG TCA ATT CCT TTA AG	1100 bp	[34]
AML1	ATC AAC TTT CGA TGG TAG GAT AGA	795 bp	[35]
AML2	GAA CCC AAA CAC TTT GGT TTC C	795 bp	[35]
NS31-GC	CGC CCG GGG CGC GCC CCG GGC GGG GCG GGG GCA CGG GGG TTG GAG GGC AAG TCT GGT GCC	300 bp	[36]
Glo1	GCC TGC TTT AAA CAC TCTA	300 bp	[36]

DGGE analysis was conducted with a D-Code Universal Mutation Detection System (Bio-Rad Laboratories). DGGE fingerprints were run on a 6% polyacrylamide gel for 15 h at a constant voltage of 70 V and at 60°C in a 30%-60% horizontal denaturant gradient (the 100% denaturant agent is 7 M urea and 40% deionized formamide). Gels were photographed with UV transillumination after SYBR- GOLD staining for 30 min. For DGGE-based microbial community analysis, Quantity One software (Bio-Rad Laboratories Inc.) was employed to calculate the Shannon-Weaver diversity index (*H*) and species evenness (*E*), while species richness (*R*) was recorded as the number of DGGE bands of each sample [37–39].

### Cloning of AMF Phylotypes and Sequencing

According to DGGE profiles, 66 bands were detected. Except 13 bands too weak to cut, 53 bands were excised for recovery and purification of DNA fragments using UNIQ-10 column DNA recovery kit (Sangon Biotech. Co., Shanghai). The purified DNA fragments were further amplified using the primer set NS31/Glo1 and products were recovered using SanPrep column DNA recovery kit (Sangon Biotech. Co., Shanghai).

PCR products were cloned into pMD19-T vector (TaKaRa Co., Dalian, China), and the clones were transformed into competent *Escherichia coli* DH5α cells (TaKaRa Co., Dalian, China) by heat shock at 42°C for 90 s. After overnight growth on Luria-Bertani plate with X-gal/IPTG (containing 100 μg/ml ampicillin) at 37°C, the white clones were picked and incubated overnight in Luria-Bertani medium (containing 100 μg/ml ampicillin) at 37°C shaken at 240 rpm. Only the clones with single band of appropriate size were selected for sequencing. Sequencing was performed commercially (Invitrogen Co., Shanghai, China). The vector sequences were eliminated using VecScreen (http://www.ncbi.nlm.nih.gov/VecScreen/) [40]. Altogether, approximately 84.9% (45/53) of sequenced clones were usable Glomeromycota sequences. Basic Local Alignment Search Tool (BLAST) searches were conducted to find sequences that shared high identity with our fungal phylotypes. Furthermore, a phylogenetic tree was constructed using Clustal X1.83 and MEGA 4.0. All sequences from our study were submitted to GenBank and the accession numbers were KP238323 to KP238367.

### Data Analysis and Statistics

All data were the means of four replicates. Principle component analysis (PCA), categorical principle component analysis (Cat-PCA, also called optimal scaling) and clustering analysis (CA) were performed using SPSS v21.0 (IBM SPSS In., Chicago). For soil chemical properties, PCA and CA were conducted to group soils of different land use types. For DGGE-based genetic data, PCA and CA were conducted according to the presence (1) or absence (0) of each particular band. Taking band matrics as analysis variables, soil physiochemical properties as supplementary variables, land use types as labeling variables, Cat-PCA was conducted to investigate the impacts of soil properties on band distribution among different soils and the relationship between soil properties and land use types [41], [42]. Biplot was constructed for visualization.

## Results

### Effects of Land Use Types on Soil Chemical Properties

Table 2 shows that the soil chemical properties of the different plots were different. Total P content, available P content and available K content were the highest in vegetable field soils and the lowest in forest land soils, with the medium in orchard soils. In contrast, total N content, soil organic matter content, total K content and available N content were the highest in forest land soils and the lowest in vegetable field soils, again with the medium in orchard soils (Table 2). These data strongly supported that high intensity of land use increased total P content, available P content and available K content, while decreased total N content, soil organic matter content, total K content and available N content. Interestingly, it seems that soil pH was not related to the intensity of land use.

**Table 2:** Chemical properties of soils as influenced by different land use types.

Soil chemical properties	Forest land	Orchard	Vegetable field
pH	4.68±0.15c	6.20±0.13a	5.60±0.03b
Soil organic matter (g/kg)	31.0±2.7a	18.7±0.7b	15.2±0.5b
Total N (g/kg)	1.11±0.09a	0.83±0.02b	0.60±0.02c
Total P (g/kg)	0.19±0.01c	0.59±0.09b	0.78±0.05a
Total K (g/kg)	13.40±1.24a	13.28±0.64a	8.97±0.09b
Available N (mg/kg)	68.45±5.92a	59.97±2.16a	40.03±1.49b
Available P (mg/kg)	1.42±0.41c	38.31±8.56b	120.01±11.19a
Available K (mg/kg)	29.97±1.03c	64.67±13.06b	138.32±13.43a

Data are presented as average ± s.e. Data followed by the same letter are not significantly different at the 5% level (Duncan’s multiple range test) for each soil chemical property.

**Fig 1:**
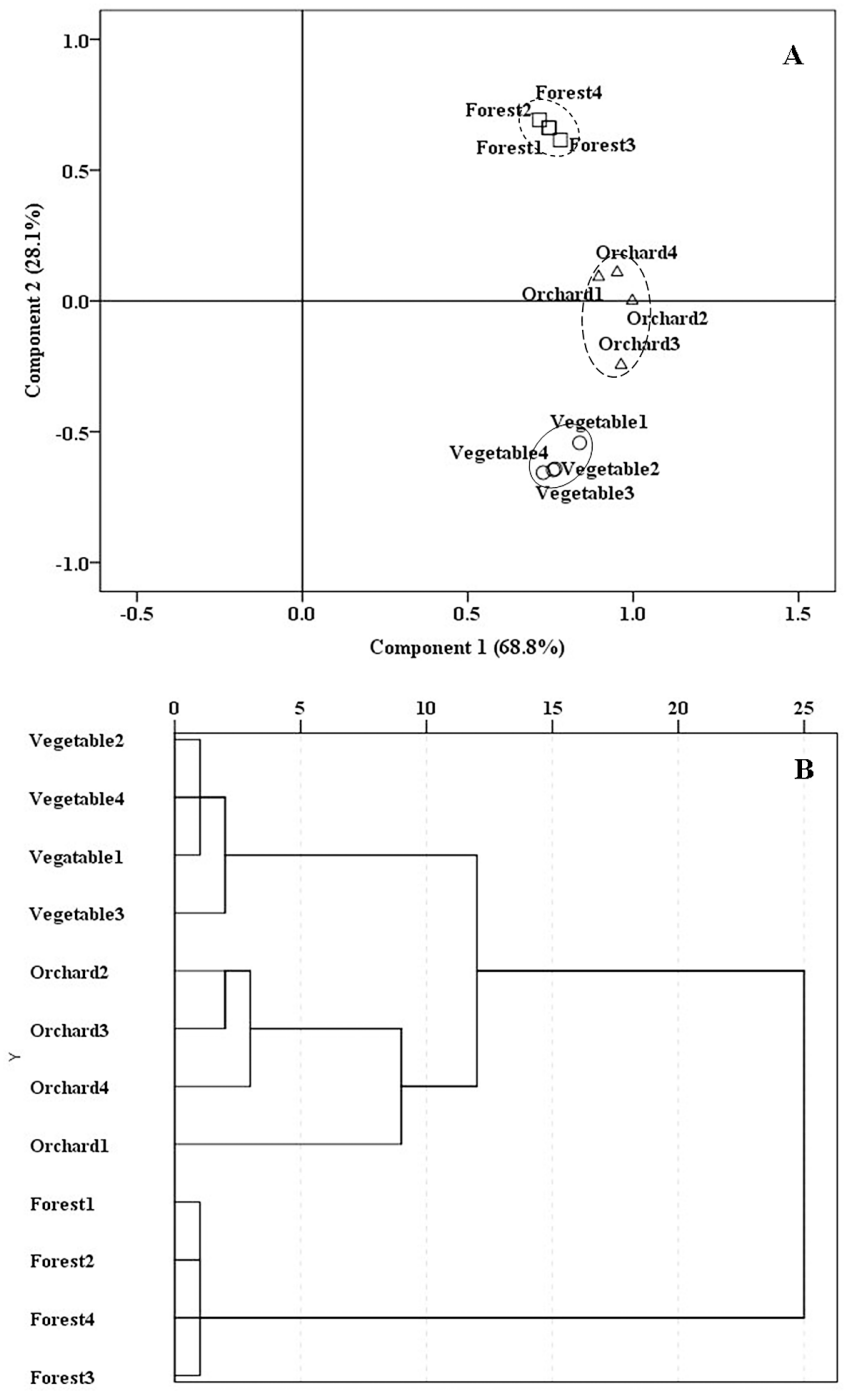
Principle component analysis (PCA) and cluster analysis (CA) of soils of different land use types based on the soil chemical properties. (A) PCA. (B) CA. Squares, triangles and circles indicate forest land soils, orchard soils and vegetable field soils, respectively. Four replicate soils of each land use type were grouped using circles.

PCA and CA demonstrated that four replicates of each land use type grouped closely (Fig 1). Three land use types were clearly separated from each other (Fig 1A), indicating their distinct soil properties. However, orchard soils were more close to vegetable field soils when compared to forest land soils (Fig 1B).

### AMF Communities in the Soils of Land Use Types

According to the spore morphology, 6, 7 and 2 morphotypes were isolated respectively from the soils of forest land, orchard, and vegetable field (S1 Fig), including *Ambispora gerdemannii*, *Claroideoglomus etunicatum*, *Funneliformis coronatum*, *F*. *verruculosum*, *F*. *caledonius*, *Rhizophagus diaphanus*, *Septoglomus deserticola*, *S*. *constrictum*, *Glomus* sp., and five other unidentified Glomeromycota sp. Despite the unidentified morphotypes, 88.9% of the identified morphotypes belong to Glomeraceae.

**Fig 2:**
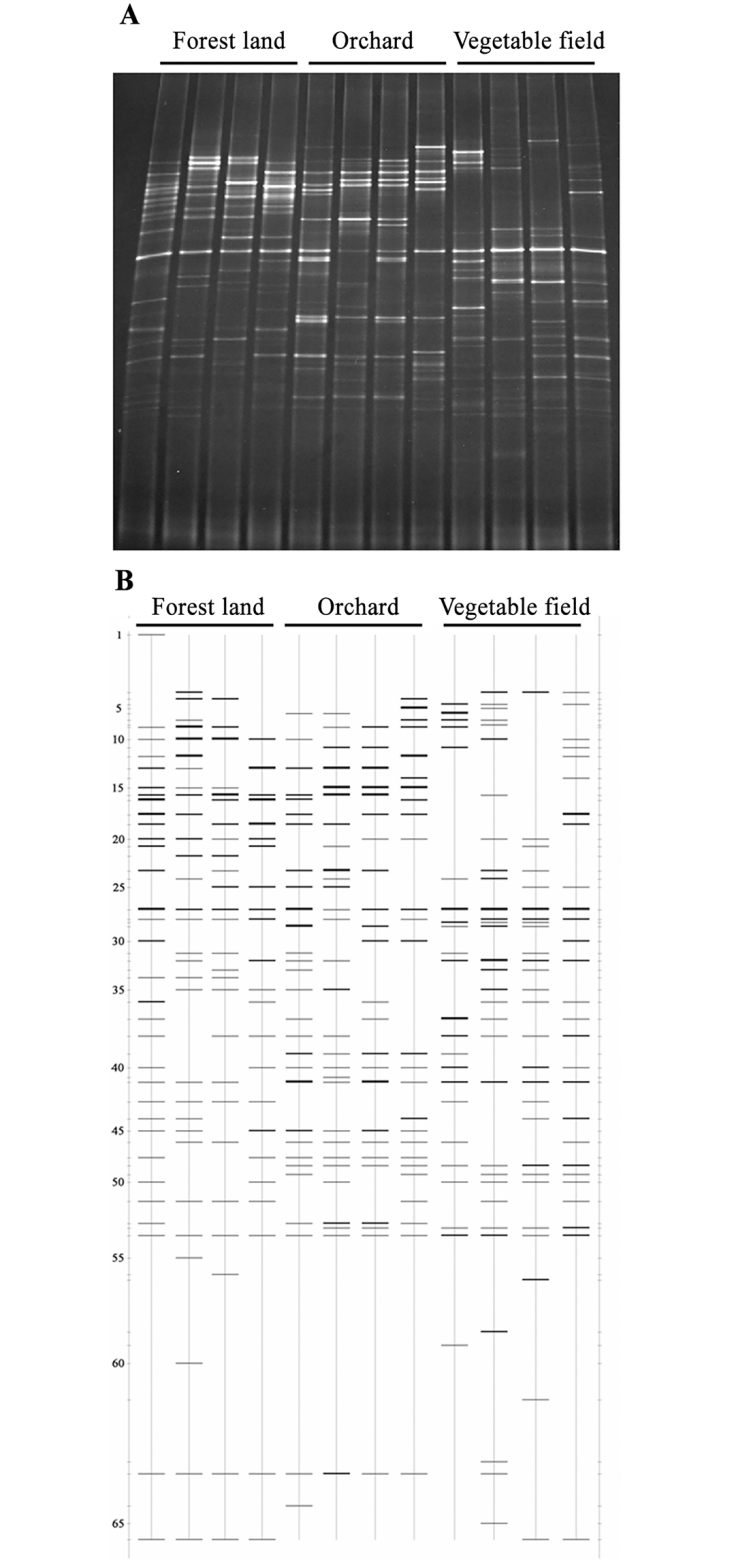
DGGE profiles and their corresponding patterns of 18S rDNA fragments of arbuscular mycorrhizal fungal (AMF) community in soils of different land use types. (A) DGGE profiles of AMF community. (B) DGGE patterns of AMF community. A total of 66 bands were detected using Quantity One software.

DGGE profiles of AMF 18S rRNA genes demonstrated that AMF community structures were much different in the soils of different land use types (Fig 2). Totally, there were 66 bands detected in the soils (Fig 2B).

The Shannon-Weaver diversity indices of AMF in soils of different land use types were similar, without significant difference between them. Similarly, land use type did not affect AMF species richness (Table 3). However, AMF species evenness in forest land soils was significantly lower than those in orchard soils and vegetable field soils (Table 3). It is worthy to note that AMF species richness of only 27.0~28.0 for each land use type in contrast to total 66 bands suggests the strong influence of land use types on AMF community composition.

**Table 3:** Community parameters of arbuscular mycorrhizal fungi in soils as influenced by different land use types.

AMF community parameters	Forest land	Orchard	Vegetable field
Shannon-Weaver diversity index (*H*)	3.17±0.05a	3.13±0.08a	3.22±0.13a
Species richness (*S*)	27.8±2.0a	26.5±1.0a	27.0±1.6a
Species evenness (*E*)	0.941±0.005b	0.957±0.004a	0.966±0.003a

Data are presented as average ± s.e. Data followed by the same letter are not significantly different at the 5% level (Duncan’s multiple range test) for each soil chemical property.

**Fig 3:**
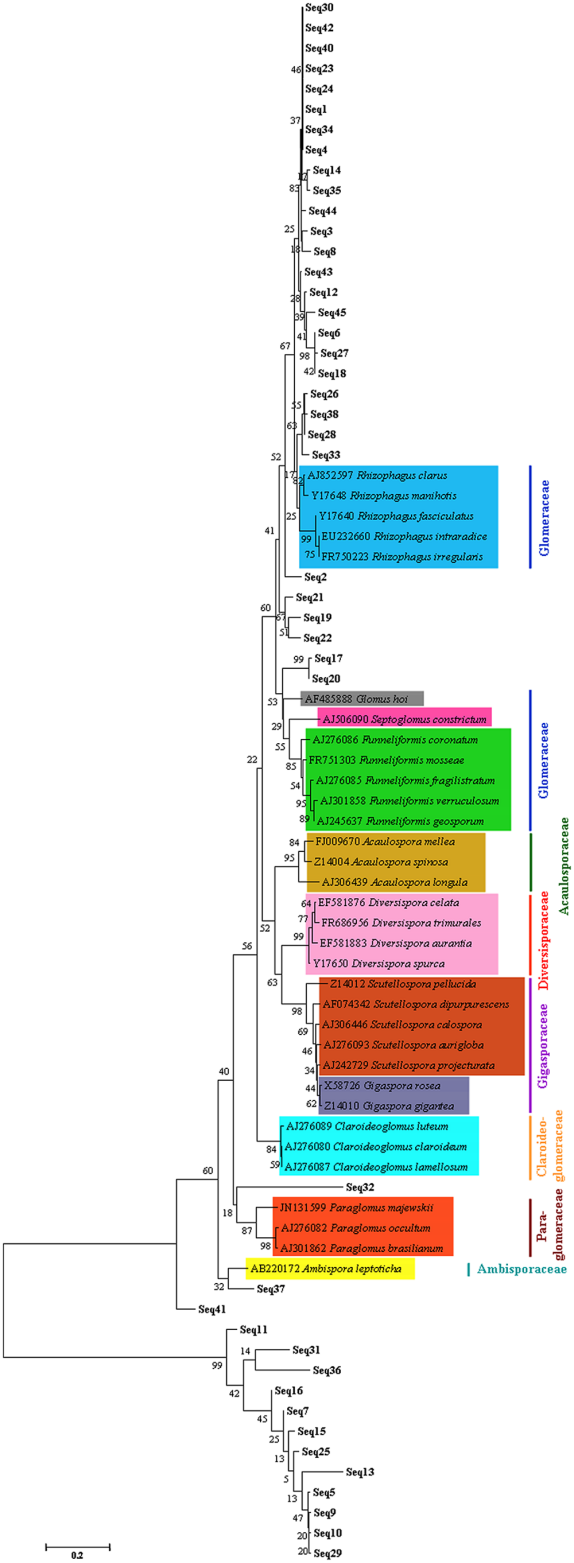
Phylogenetic tree of AMF species in the investigated soils based on the fungal 18S rDNA fragments. All the named species as reference sequences appear with the scientific names of recent taxonomy (http://schuessler.userweb.mwn.de/amphylo/).

In total, 53 out of 66 bands were excised for sequencing, and 45 sequenced bands were related (87%-100% similarity) to AMF 18S rRNA gene already deposited in the GenBank database. The results of sequence identification using the BLAST-X algorithm are shown (S1 Table). The phylogenetic tree clearly indicates that 29 AMF sequences (64.4%) belong to Glomeraceae, 1 (2.2%) belongs to Paraglomeraceae and 1 (2.2%) belongs to Ambisporaceae. The other 13 AMF sequences can not be assigned to the properly identified (mostly cultured) species (Fig 3).

**Fig 4:**
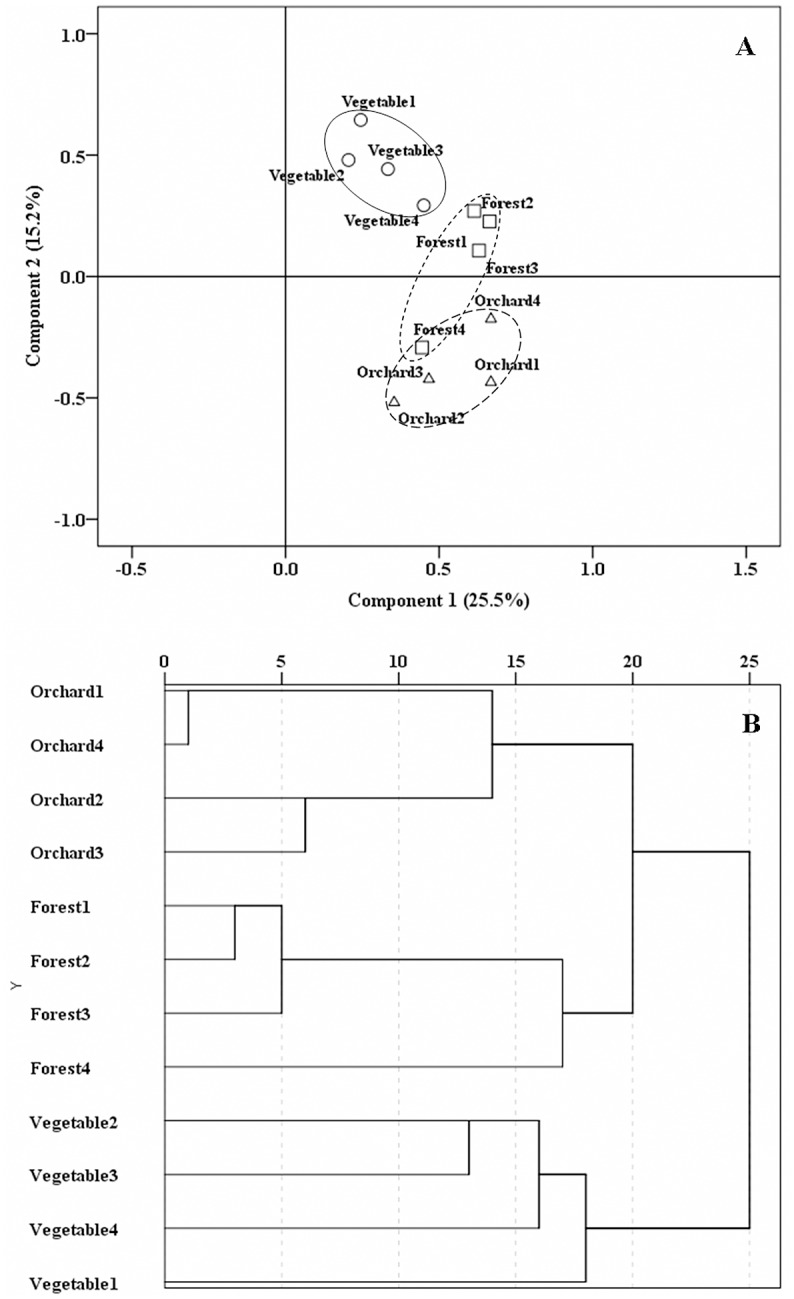
Principle component analysis (PCA) (A) and cluster analysis (CA) (B) of arbuscular mycorrhizal fungal (AMF) community in soils of different land use types based on DGGE profiles of fungal 18S rDNA fragments. Squares, triangles and circles indicate forest land soils, orchard soils and vegetable field soils, respectively. Four replicate soils of each land use type were grouped using circles.

According to the DGGE profiles, four replicate soils of each land use pattern grouped well as revealed by PCA (Fig 4A). AMF community structure in forest soils was more similar to that in orchard soils than to that in vegetable field soils, which was also supported by clustering analysis (Fig 4B).

### Effect of Land Use Types on AMF Community Composition as Revealed by Cat-PCA

**Fig 5:**
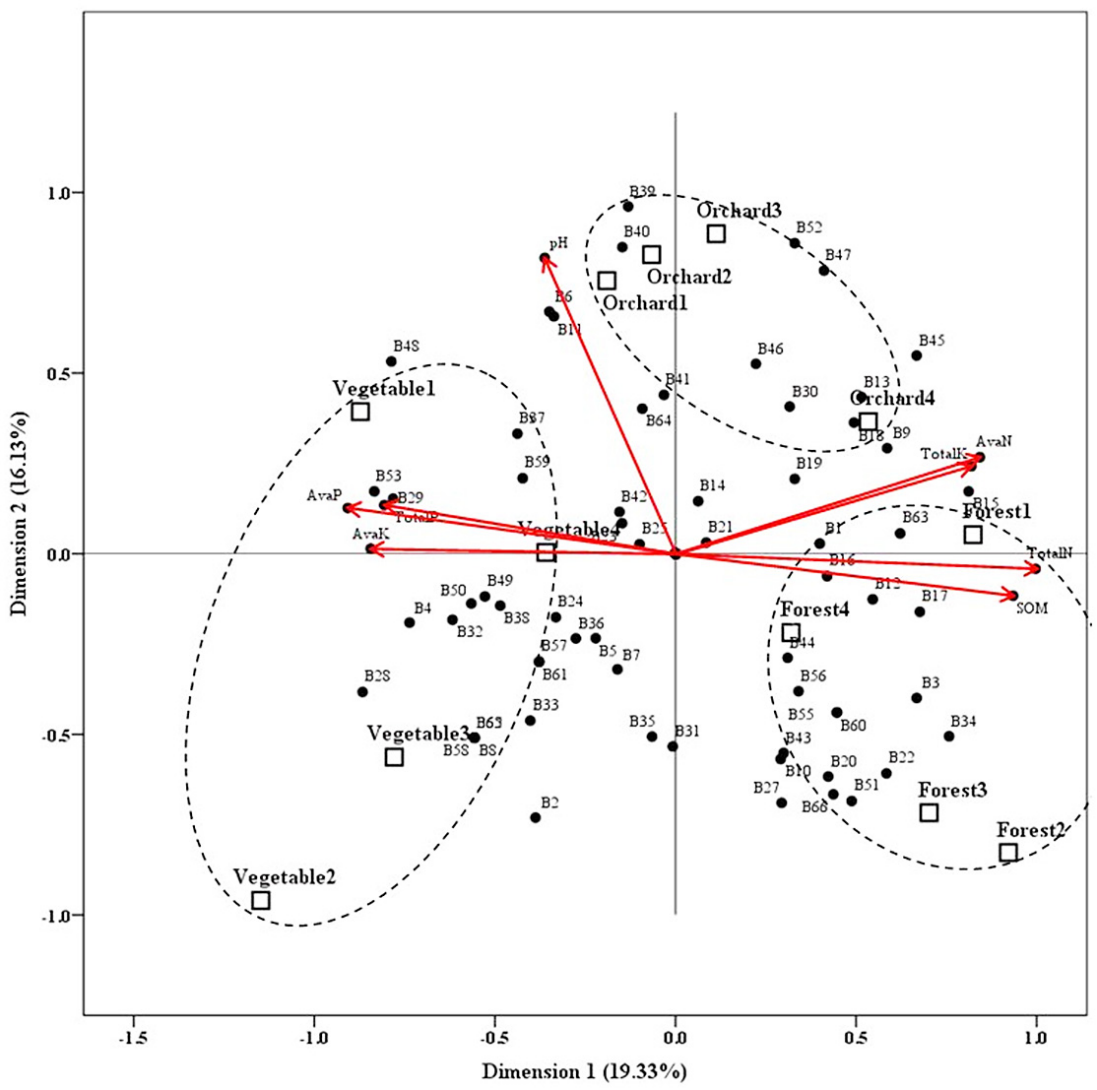
Category principle component analysis (Cat-PCA) biplot. The relationship between soils of different land use types (squares) and arbuscular mycorrhizal fungal (AMF) phylotypes (solid circles), soil chemical properties (solid circles with red arrows) are encompassed. For fungal phylotype, 1 and 0 were used to represent presence and absence of particular species in each soil sample according to DGGE profiles. Four replicate soils of each land use type were grouped using dash-lined circles. B*n* (*n* = 1~66) indicates AMF phylotype corresponding to DGGE band as shown in Fig 2.

To investigate the influence of soil chemical properties on AMF community, Cat-PCA was performed on the basis of DGGE profiles. Eight soil properties can be categorized into 3 types, with total N content, soil organic matter content, total K content and available N content positive to X-axis, available P content, available K content and total P content negative to X-axis, while pH less related to X-axis (Fig 5). According to the distance from the coordinate origin, total N content, soil organic matter content and available P content ranked the first three soil properties, indicative of the three most influential factors.

When three land use types were grouped, they separated well (Fig 5). Forest land soils located on the right part of the plot, in good accordance with total N content and soil organic matter content, indicating that forest land soils were typical of high contents of total N and soil organic matter, which is supported by Table 2. Similarly, vegetable field soils located on the left part of the plot, in good accordance with available P content, available K content and total P content, indicating that vegetable field soils were typical of high contents of available P, available K and total P, which is supported by Table 2.

All 66 bands (e.g. putative AMF phylotypes) scattered in the plot, and some of them located closely to soil chemical properties or land use types (Fig 5). For example, B29, B53, B4, B50, B49, B32, B38 are close to available P content, available K content and total P content, suggesting that these AMF phylotypes may prefer to appear in soils with high contents of available P, available K and total P (such as vegetable field soils in this study). B3, B34, B60, B22, B12, B17 locates closely to forest land soils, suggesting that these AMF phylotypes may be present in forest ecosystems. More interestingly, some “generalists” and “specialists” AMF stains were also demonstrated, e.g. B26 and B54 present in all soils while B4 and B28 present only in vegetable field soils (Fig 2).

## Discussion

### Domination of Glomeraceae in Studied Soils

In this study, AMF communities in soils of three land use types were investigated. Sequencing and BLAST analysis indicate that most (>64%) AMF phylotypes belong to the Glomeraceae (S1 Table), and meanwhile the AMF identification based on spore morphology also supported it. In other investigation, similar result was obtained. Zhang et al. isolated 40 AMF taxa from rhizosphere soils of pteridophytes, and indicated that 80% taxa belonged to *Glomus*, the largest genus in the Glomeraceae [43].

It is well established that the Glomeraceae, the largest family of AMF, can adapt to diverse ecosystems [14]. This can be a reason why the Glomeraceae dominates in the sites of our study. More importantly, in our study, the dominant plants in the understory of forest land and in covering plants in orchard were ferns (*Dicranopteris*) [28]. As for vegetable field, it has been previously dominated by fern plants for decades. The domination by fern plants in these soils probably determines the AMF community composition to a large degree. A large body of investigation indicates that the Glomeraceae is the most associated AMF family with fern plants [43–45]. Kovács et al. [44] found that most of the AMF detected in *Botrychium virginianum* belonged to *Glomus* group A, although several AMF lineages showing similarities with *Scutellospora* (Gigasporaceae) also colonized roots. In the investigation of AMF status of ferns collected from Eastern and Western Ghats regions in India, Muthukumar and Prabha [45] reported a total of nine AMF spore morphotypes, including *Glomus*, *Claroideoglomus*, *Funneliformis* and *Rhizophagus*, all of which belong to the Glomeraceae. This is in well agreement with our result. Differently, we identified AMF based on the sequence of a small DNA fragment, which was amplified with the primer set NS31/Glo1. This primer set was demonstrated to amplify all genera (except *Septoglomus*) from the Glomeraceae and even genera from the families of Ambisporaceae, Claroideoglomeraceae, Gigasporaceae, Acaulosporaceae, Paraglomeraceae [46]. In a field investigation, 96% of *Angiopteris lygodiifolia* and 95% of *Osmunda japonica* gametophytes contained AMF hyphae, and molecular analysis based on SSU rDNA revealed that all these AMF belonged to *Glomus* group A [47].

### Effect of Land Use Types on AMF Community Composition

We selected three land use types and established four sampling plots for each land use type. However, for each land use type, due to the small distance among individual plot, the difference in AMF community composition in each plot represented the within-locality variation. Despite of this, data in our study indicate that land use types affected the AMF community composition, as revealed by DGGE profile (Fig 2) and BLAST analysis (S1 Table), although Shannon-Weaver diversity index and species richness were not affected. For example, B4 and B28 were present only in vegetable field soils, while B13 and B15 were absent only in these soils. Similar to our results, results of González-Cortés et al. [48] suggest that the impact of land use change is greater on the community composition than on the richness of AMF. The different AMF community composition modified by land use types has been reported elsewhere. Helgason et al [49] revealed the different AMF community in adjacent soils of wood land and arable land. Bedini et al. [50] indicated that different land use types (maize monoculture, grassland and poplar grove) affected not only AMF population (spore number) but also AMF functioning (GRSP content). Land use types significantly affected glomalin concentrations, with native forest soils having the highest concentrations when compared to the soils of an afforested system and an agricultural field [51].

Interestingly, most investigation found that increased land use intensity is unbeneficial to AMF development. The numbers of AMF spores and species were highest in the grasslands, lower in the low- and moderate-input arable lands, and lowest in the lands with intensive continuous maize monocropping [23]. They concluded that the increased land use intensity was correlated with a decrease in AMF species richness and with a preferential selection of species that colonized roots slowly but formed spores rapidly. Based on the spore morphology, Oehl et al. [52] indicated that the diversity and community composition of AMF showed strong dependence on land use intensity. In these AMF communities, several species were associated with a specific land use type while others could be considered as “generalists” colonizing all soils. This result is similar to that in our study, demonstrating the existence of some “specialists” and some “generalists”. Spore density and species richness were generally higher in the natural savannas and under yam than at the other cultivated sites and lowest under the intensively managed cotton [27]. Despite of data from morphological study, genetic data also supported this point. Using pyrosequencing approach, Lumini et al. [53] demonstrated that the environments with low inputs (pasture and covered vineyard) showed a higher AMF biodiversity than those subjected to human input (managed meadow and tilled vineyard). In contrast to this point, some studies indicated that high intensity of land use did not change or even increased AMF diversity or species richness. Jefwa et al. [26] reported that AMF species diversity and richness were maintained unchanged despite dramatic changes in land use types including indigenous forest, planted forest and croplands with coffee, maize, horticulture or napier, although some AMF species showed preference for either cropped or non-cropping systems. Stürmer and Siqueira [25] even found that mean AMF species richness in crop, agroforestry, young and old secondary forest sites was twice that in pristine forest and pasture in Western Brazilian Amazon following the conversion of pristine forest into distinct land uses. They argued that practices adopted in this region helped maintain a high AMF diversity.

### Impact of Land Use Types on AMF Community via Soil Chemical Properties

We explored the relationships between AMF composition, soil chemical properties and land use types using Cat-PCA. Three land use types were grouped well and were also in well accordance with soil properties, e.g. forest land soils (with high contents of SOM, total N, total K and available N but with low content of total P, available P and available K) *versus* vegetable field soils (*vice versa*). Furthermore, these soil properties are well associated with some AMF phylotypes in our study. For example, B29, B53 are closely associated with high contents of available P, available K and total P. These data strongly suggest that land use types affect AMF community composition *via* soil chemical properties, which has been demonstrated in diverse ecosystems [52], [54], [55].

It is worthy to note that AMF phylotypes in vegetable field soils may be of ecological significance. Douds and Schenck [56] reported that *Rhizophagus intraradices* (formerly known as *Glomus intraradices*) is tolerant to P levels. In a study investigating the AMF diversity in greenhouse soils, *Acaulospora excavata*, *A*. *rehmii*, *Glomus aggregatum*, *Claroideoglomus claroideum* (formerly known as *G*. *claroideum*), *Claroideoglomus etunicatum* (formerly known as *G*. *etunicatum*) and *Funneliformis mosseae* (formerly known as *G*. *mosseae*) appeared in soils with 151 mg/kg available N, 59 mg/kg available P and 291 mg/kg available K, and *A*. *nicolsonii*, *C*. *claroideum*, *C*. *etunicatum* and *F*. *mosseae* appeared in soils with 178 mg/kg available N, 75 mg/kg available P and 355 mg/kg available K [57]. In our study, the available P and available K were as high as 120 mg/kg and 138 mg/kg in vegetable field soils. The nutritional function offered by AMF is less important in these circumstances, and thus their novel ecological functions and the corresponding consequence are to be explored.

## Supporting Information

S1 Fig Some AMF spores isolated from the soils of forest land, orchard, vegetable field. (DOC)

S1 Table Identification by NCBI BLASTX (first hit and most similar sequences from known arbuscular mycorrhizal fungi) of 18S rRNA gene sequences retrieved from DGGE bands. The number of each band corresponds to that as shown in Fig 2.
(DOC)
