# Supplementary material for: Differences in Arbuscular Mycorrhizal Fungal Community Composition in Soils of Three Land Use Types in Subtropical Hilly Area of Southern China
Source: PLoS One. 2015 Jun 24;10(6):e0130983. doi: 10.1371/journal.pone.0130983 (PMC4479462; doi:10.1371/journal.pone.0130983)

**S1A.** Some AMF spores isolated from the forest land soils. A: *Steptoglomus deserticola*; B: *Funneliformis coronatum*; C: *Funneliformis caledonius*; D: *Septoglomus constrictum*; E: Unidentified Glomeromycota sp.; F: *Glomus* sp.


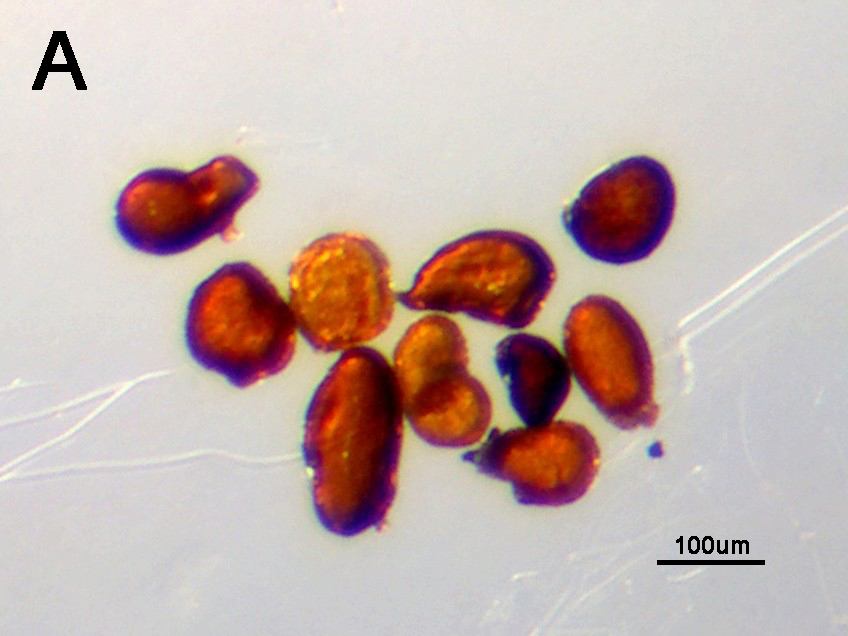

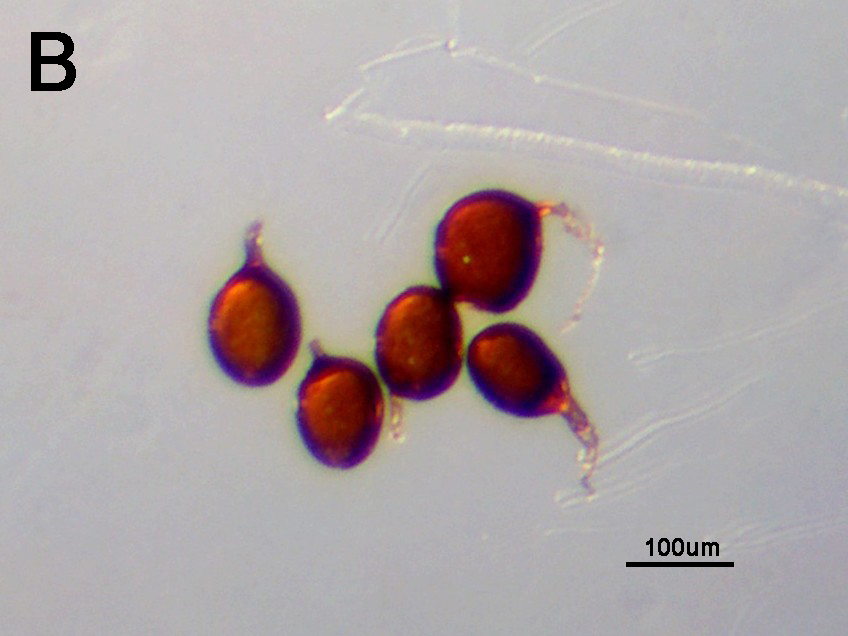


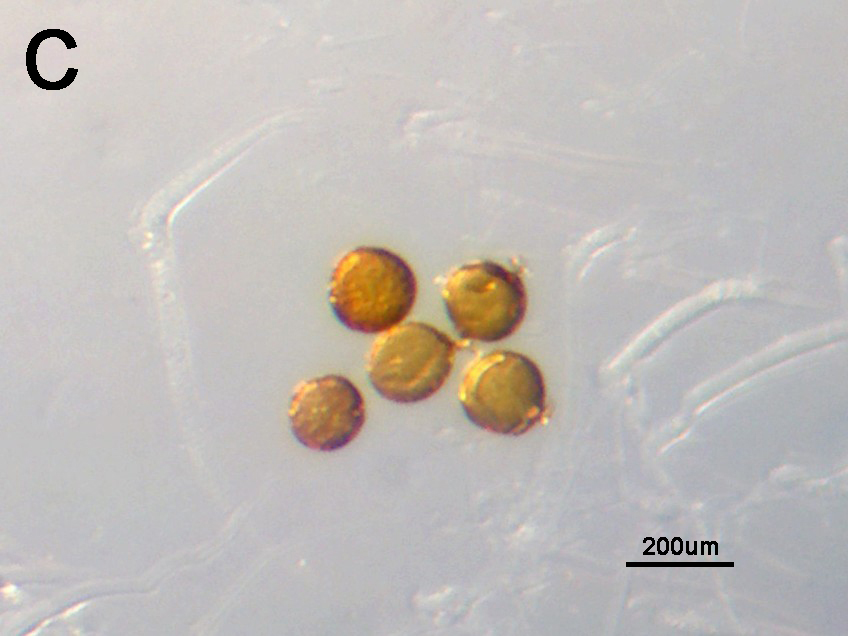

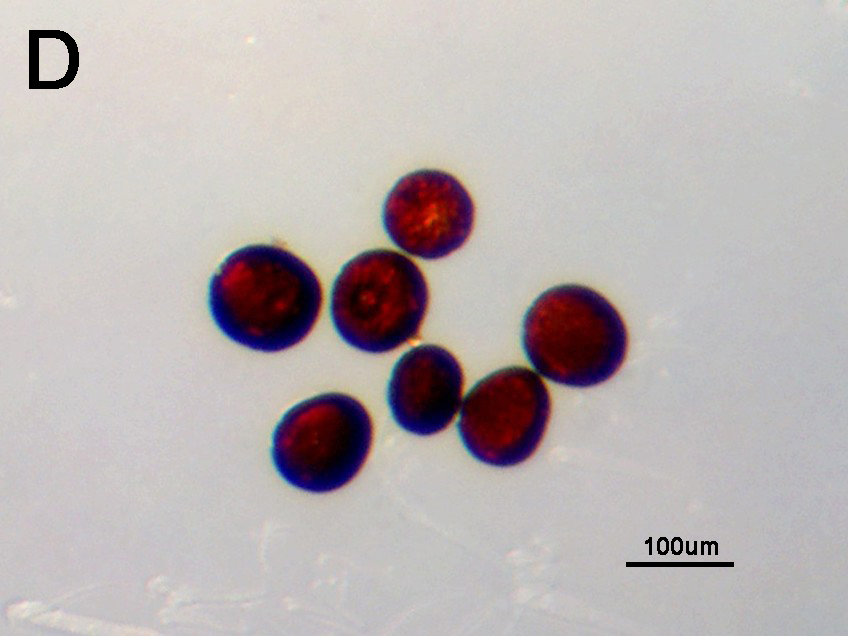


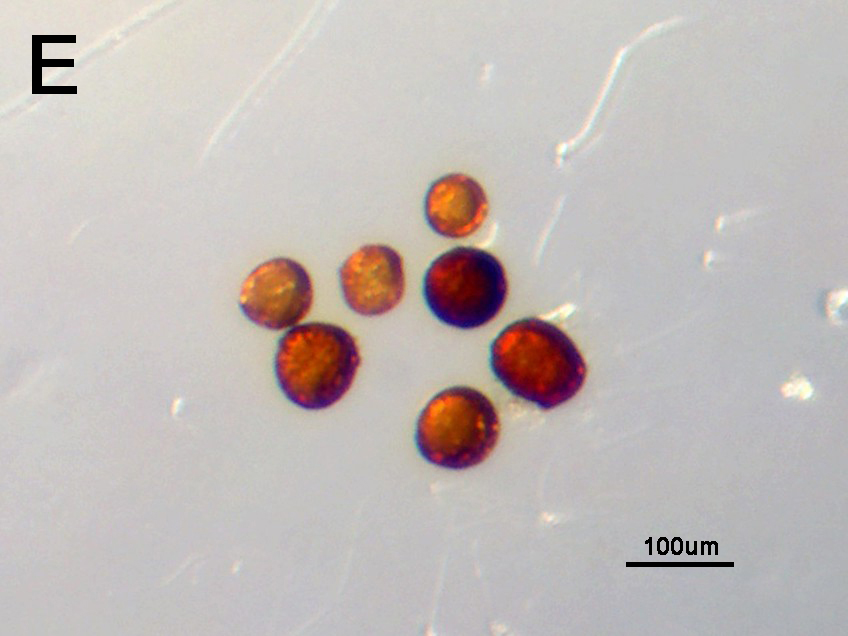

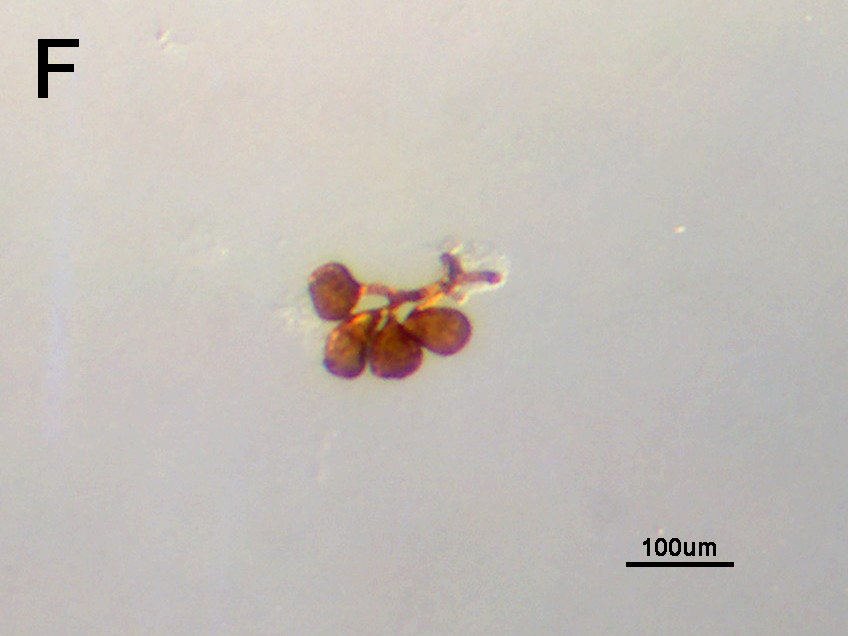


**S1B.** Some AMF spores isolated from orchard soils. A: *Rhizophagus diaphanus*; B: *Funneliformis verruculosum*; C: *Septoglomus constrictum*; D: *Claroideoglomus etunicatum*; E: Ambispora gerdemannii; F: Unidentified Glomeromycota sp.; G: Unidentified Glomeromycota sp.


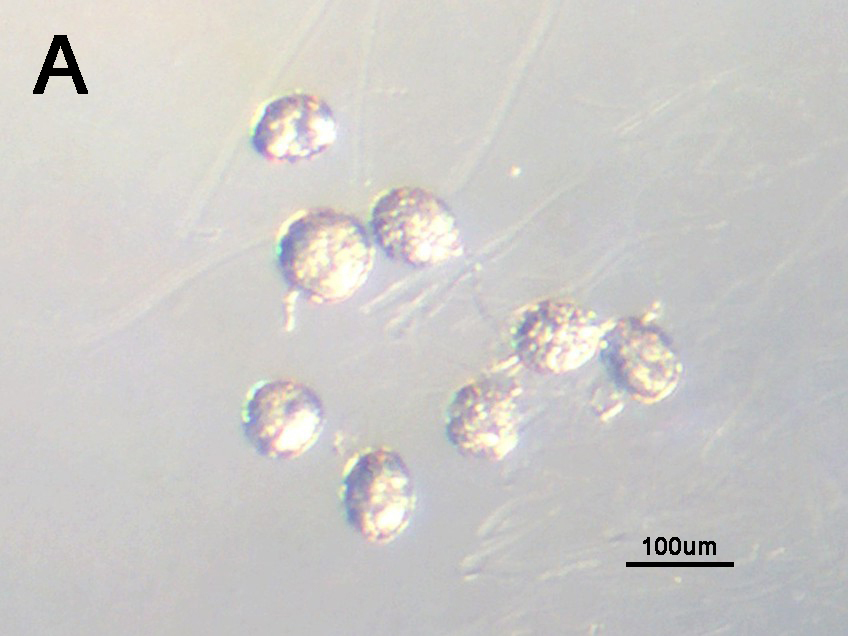

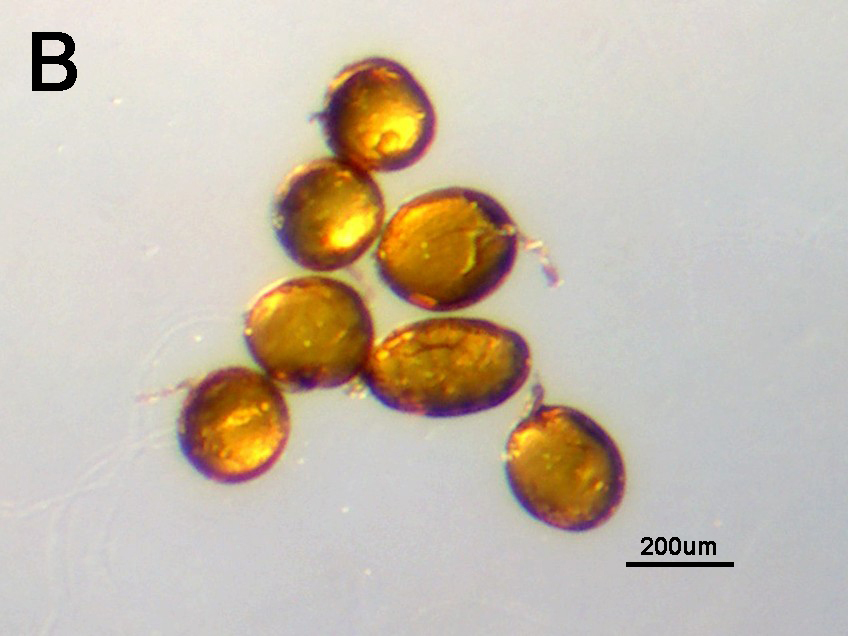


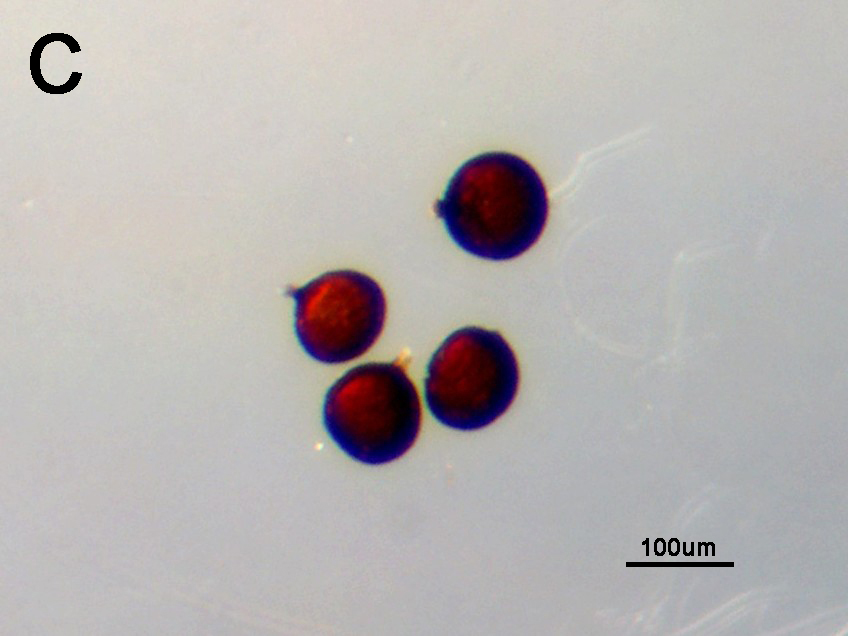

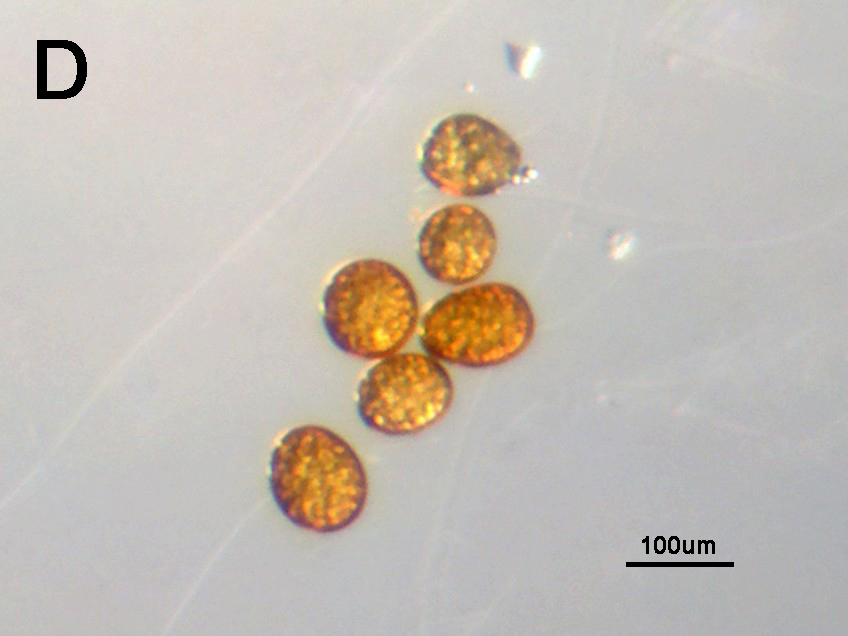


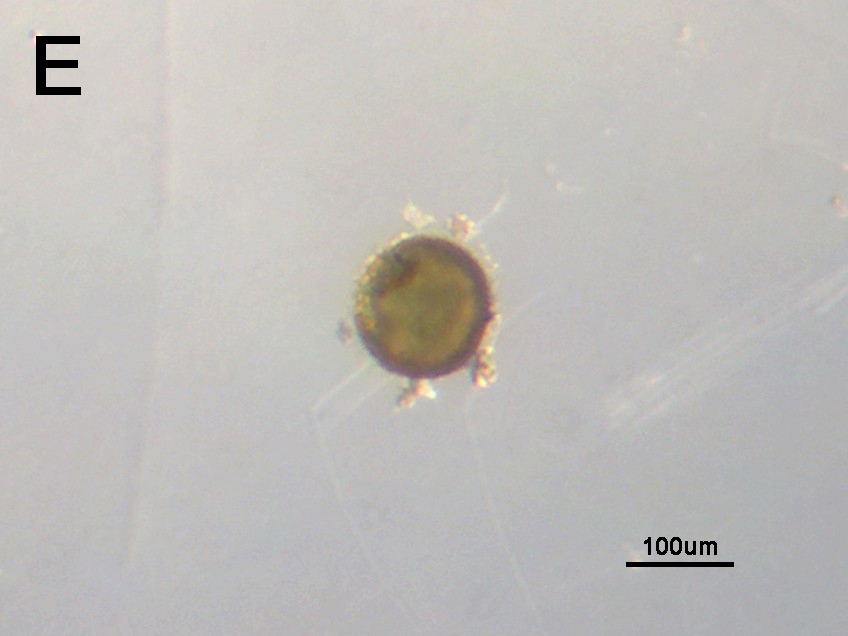

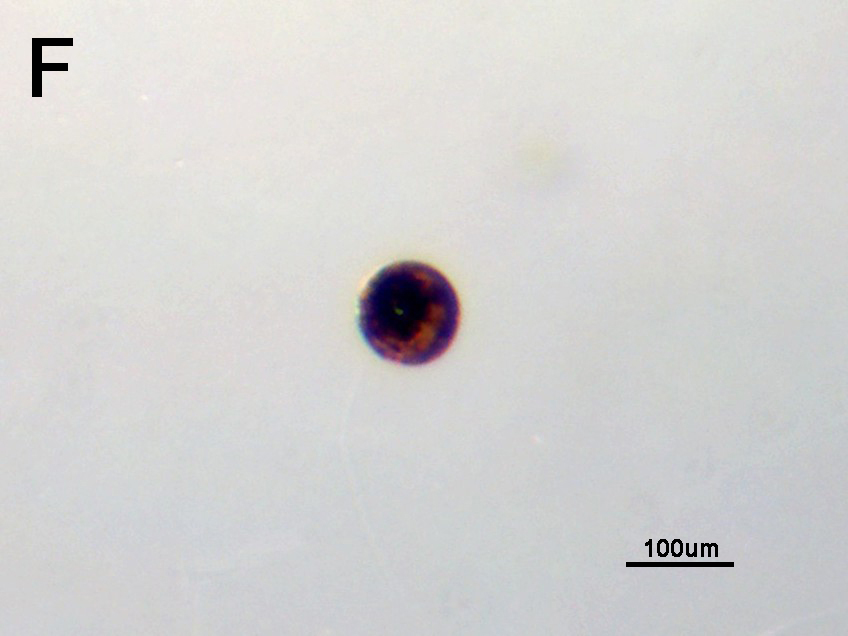


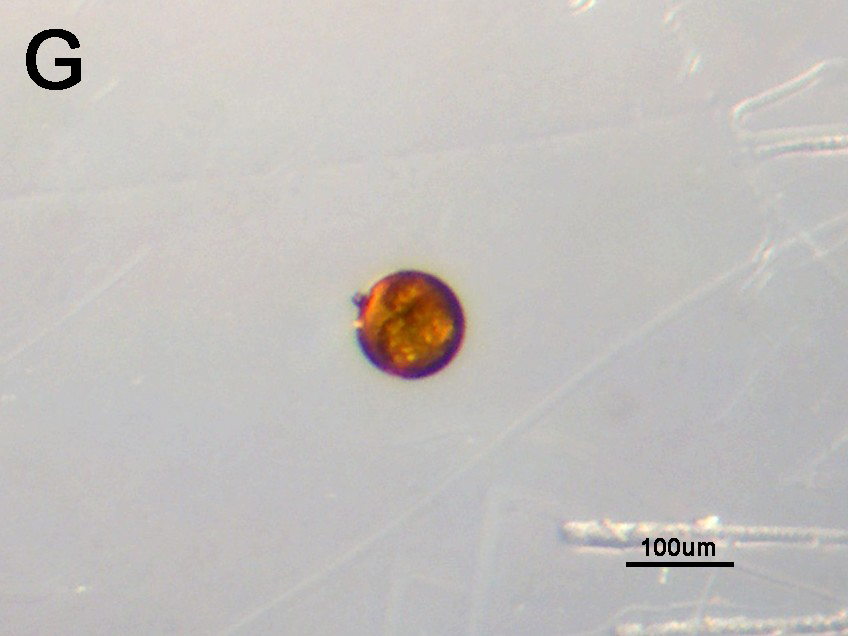


**S1C.** Some AMF spores isolated from the vegetable field soils. A: Unidentified Glomeromycota sp.; B: Unidentified Glomeromycota sp.


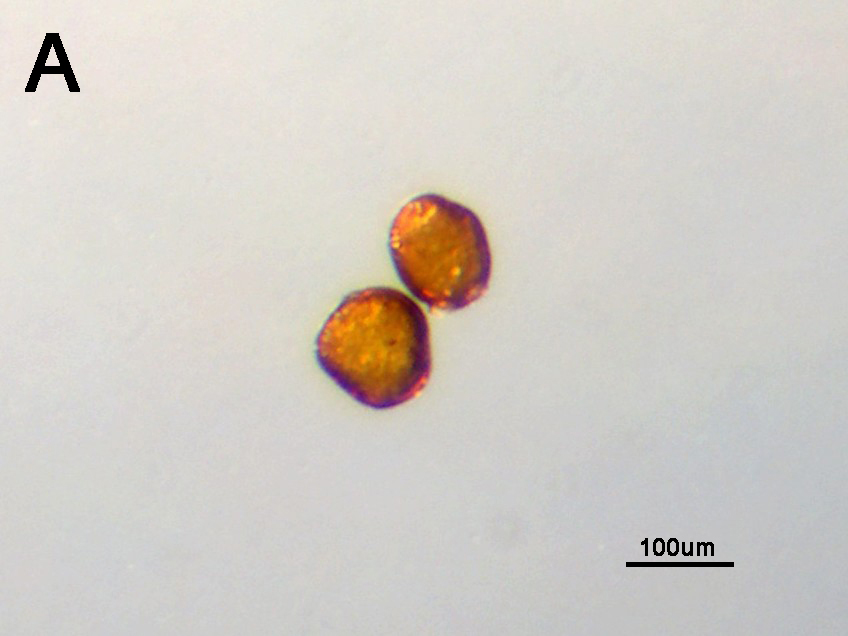

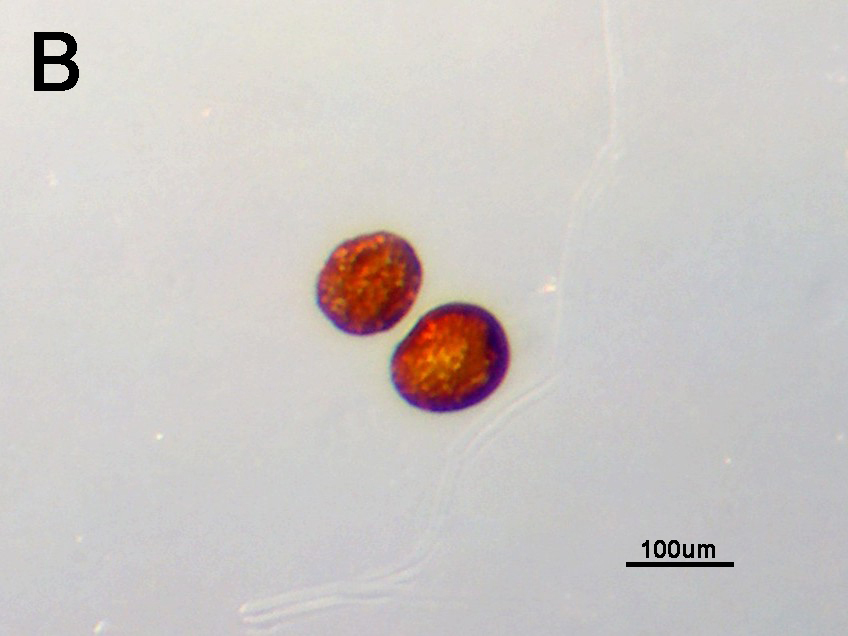

Supplement: S1 Fig — (DOC) [file pone.0130983.s001.doc]
