# Supplementary material for: Differences in Arbuscular Mycorrhizal Fungal Community Composition in Soils of Three Land Use Types in Subtropical Hilly Area of Southern China
Source: PLoS One. 2015 Jun 24;10(6):e0130983. doi: 10.1371/journal.pone.0130983 (PMC4479462; doi:10.1371/journal.pone.0130983)
Supplement: S1 Table — The number of each band corresponds to that as shown in Fig 2. (DOC) [file pone.0130983.s002.doc]

**S1 Table. Identification by NCBI BLASTX (first hit and most similar sequences from known arbuscular mycorrhizal fungi) of 18S rRNA gene sequences retrieved from DGGE bands.** The number of each band corresponds to that as shown in Fi. 2.

| Band | Closest match (first hit) | Identity | Accession Number |
| --- | --- | --- | --- |
| 1 | *Glomus* sp. PSAMG2 gene for 18S rRNA, partial sequence, clone: SacAM-3 | 98% | AB178730.1 |
| 2 | *Glomus* sp. Glo2 isolate 2st3 small subunit ribosomal RNA gene, partial sequence | 93% | AY129631.1 |
| 4 | *Glomus* sp. PSAMG2 gene for 18S rRNA, partial sequence, clone: SacAM-2 | 96% | AB178729.1 |
| 6 | *Glomus* sp. PSAMG2 gene for 18S rRNA, partial sequence, clone: SacAM-1 | 98% | AB178728.1 |
| 7 | *Glomus* sp. Glo3 partial 18S rRNA gene, clone epw4_15 | 96% | AJ715998.1 |
| 8 | *Glomus* sp. spore-6-B3 18S ribosomal RNA gene, partial sequence | 94% | JQ182770.1 |
| 9 | *Glomus* mycorrhizal symbiont of *Marchantia foliacea* 18S rRNA gene (partial) and ITS1 (partial), isolate Ross II, clone 2 | 96% | AJ699068.1 |
| 10 | *Glomus* sp. Glo30 isolate 2sf9 small subunit ribosomal RNA gene, partial sequence | 95% | AY129611.1 |
| 11 | *Glomus* sp. Glo3b isolate 1bt3 small subunit ribosomal RNA gene, partial sequence | 94% | AY129592.1 |
| 12 | *Glomus* mycorrhizal symbiont of *Marchantia foliacea* 18S rRNA gene (partial) and ITS1 (partial), isolate Ross I, clone 1 | 94% | AJ699067.1 |
| 13 | *Glomus* sp. 0702-3 18S ribosomal RNA gene, partial sequence | 93% | EU332739.1 |
| 14 | *Glomus* sp. Glo49 clone bpr6 small subunit ribosomal RNA gene, partial sequence | 94% | AY512364.1 |
| 15 | *Glomus* sp. VeGlo18 partial 18S rRNA gene, clone m1-9 | 95% | FN429381.1 |
| 16 | *Glomus* sp. Glo3 clone T1f9c599 small subunit ribosomal RNA, partial sequence | 95% | AF481678.1 |
| 17 | *Glomus* sp. PSAMG1 gene for 18S rRNA, partial sequence, clone: RhyAM-1 | 96% | AB178727.1 |
| 18 | Rhizophagus clarus 18S rRNA gene, isolate UFPE08 | 95% | AJ852597.1 |
| 19 | Funneliformis geosporum partial 18S rRNA gene, isolate Att1495-0, clone pMK112-3 | 93% | FR772325.1 |
| 20 | Sclerocystis sinuosa 18S rRNA gene | 94% | AJ133706.1 |
| 21 | *Glomus* sp. Glo2 clone 853DNAT2 small subunit ribosomal RNA gene, partial sequence | 96% | AF481554.1 |
| 22 | *Glomus* sp. Glo9 isolate C_Ru_5.5 small subunit ribosomal RNA gene, partial sequence | 93% | AF074355.1 |
| 23 | *Glomus* sp. Glo37 small subunit ribosomal RNA gene, partial sequence | 100% | AY129588.1 |
| 25 | *Glomus* sp. MO-G5 partial 18S rRNA gene, clone cMO63.1 | 97% | AJ496085.1 |
| 26 | *Glomus* sp. Glo3 clone atn8 small subunit ribosomal RNA gene, partial sequence | 96% | AY512357.1 |
| 27 | *Glomus* sp. Glo3 clone atr6 small subunit ribosomal RNA gene, partial sequence | 96% | AY512354.1 |
| 28 | *Glomus* sp. Glo3 clone T2f11c18 small subunit ribosomal RNA, partial sequence | 95% | AF481697.1 |
| 29 | *Glomus* sp. Glo30 isolate 1bt7 small subunit ribosomal RNA gene, partial sequence | 98% | AY129606.1 |
| 30 | *Funneliformis mosseae* partial 18S rRNA gene, clone IMA1/1-31 | 94% | FR751295.1 |
| 31 | *Glomus* sp. Glo30 isolate M1 small subunit ribosomal RNA gene, partial sequence | 97% | AY129634.1 |
| 32 | *Glomus* sp. VeGlo10 partial 18S rRNA gene, clone Mel2-9 | 93% | FN429105.1 |
| 33 | *Glomus* sp. Glo3 clone 56 small subunit ribosomal RNA gene, partial sequence | 96% | AY273582.1 |
| 34 | *Glomus* sp. NBR8.7 clone NBR8-7-29 18S small subunit ribosomal RNA gene, partial sequence | 92% | EF136905.1 |
| 35 | Glomeromycota sp. MIB jd152 voucher M.I. Bidartondo:jd152 clone 1 18S ribosomal RNA gene, partial sequence | 97% | JF414172.1 |
| 36 | *Glomus* sp. 0904-1 18S ribosomal RNA gene, partial sequence | 97% | EU332734.1 |
| 37 | *Glomus* sp. Glo3c clone 39 small subunit ribosomal RNA gene, partial sequence | 96% | AY273592.1 |
| 38 | *Glomus* sp. VeGloF partial 18S rRNA gene, clone Mel2-5 | 94% | FN429104.1 |
| 39 | *Ambispora gerdemannii* partial 18S rRNA gene | 90% | AM400227.1 |
| 41 | *Glomus* sp. UY1184.10 small subunit ribosomal RNA gene, partial sequence | 87% | AF485861.1 |
| 45 | *Glomus* arbuscular mycorrhizal symbiont of *Voyria corymbosa* 18S rRNA gene, *Voyria corymbosa* sample Q | 97% | AJ430853.1 |
| 46 | Uncultured *Glomus* clone 13 18S ribosomal RNA gene, partial sequence | 92% | JF432012.1 |
| 48 | *Glomus* sp. Glo3c clone 89 small subunit ribosomal RNA gene, partial sequence | 96% | AY273591.1 |
| 53 | *Ambispora leptoticha* gene for 18S rRNA, isolate: MAFF520055, sub_clone: f3b21 | 93% | AB047303.1 |
| 54 | *Glomus* sp. Glo3c clone 02 small subunit ribosomal RNA gene, partial sequence | 96% | AY273590.1 |
| 55 | *Glomus* sp. Glo3 clone 48 small subunit ribosomal RNA gene, partial sequence | 95% | AY273588.1 |
| 57 | *Glomus* sp. Glo3 clone 31 small subunit ribosomal RNA gene, partial sequence | 93% | AY273586.1 |
| 58 | *Glomus* sp. Glo3 clone 37 small subunit ribosomal RNA gene, partial sequence | 93% | AY273587.1 |
